# Breaking the silence: examining campus sexual violence and the psychological effects on women survivors

**DOI:** 10.1590/1980-220X-REEUSP-2026-0155en

**Published:** 2026-08-24

**Authors:** Umi Muzayanah, Siti Muawanah, Nur Laili Noviani, Arif Gunawan Santoso, Dwi Martiningsih

**Affiliations:** 1 National Research and Innovation Agency, Research Organization for Social Sciences and Humanities, Research Center for Religion and Belief, Jakarta, Indonesia.; 2 Universitas Islam Negeri Salatiga, Doctoral Program, Postgraduate School, Islamic Education, Salatiga, Indonesia.

**Keywords:** Universities, Sexual Violence, Survivors, Women, Disclosure, Universidades, Delitos Sexuais, Sobreviventes, Mulheres, Revelação

## Abstract

**Objective:**

To explore sexual violence in Indonesian higher education and the psychological impact on women who intend to speak up.

**Method:**

This mixed-methods study combined an online survey of 472 respondents, including students, lecturers, officials, and staff from two universities, with follow-up interviews to enrich and validate the survey.

**Results:**

Sexual violence occurred in various forms on campus, with 12% of respondents reporting victimization and 18% choosing not to disclose the experiences. Some contributing factors to sexual violence on campuses include peer pressure, power abuse, lower moral values, and the ability to conduct sexual violence. The victims dealt with significant psychological problems, such as trauma, fear, depression, a desire to self-harm, and a decline in academic performance.

**Conclusion:**

Campuses must have comprehensive interventions for sexual violence survivors to create a safer and more responsive academic environment.

## RESUMO

**Objetivo:**

Explorar a violência sexual no ensino superior na Indonésia e seu impacto psicológico em mulheres que desejam denunciá-la.

**Método:**

O estudo de métodos mistos combinou uma pesquisa online com 472 respondentes, incluindo estudantes, docentes, funcionários e colaboradores de duas universidades, com entrevistas de acompanhamento para enriquecer e validar os resultados da pesquisa.

**Resultados:**

Os resultados revelam que a violência sexual ocorre de diversas formas no campus, com 12% dos respondentes relatando vitimização e 18% optando por não divulgar suas experiências. Este estudo também constatou que alguns fatores que contribuem para a violência sexual nos campi incluem pressão dos pares, abuso de poder, valores morais baixos e a capacidade de praticar violência sexual. Algumas das vítimas relataram ter que lidar com problemas psicológicos significativos, como trauma, medo, depressão, desejo de automutilação e queda no desempenho acadêmico.

**Conclusão:**

O estudo destacou que os campi devem ter intervenções abrangentes para sobreviventes de violência sexual a fim de criar um ambiente acadêmico mais seguro e acolhedor.

## INTRODUCTION

The increasing prevalence of sexual violence on campuses is deeply concerning, reflecting a contradiction amid global commitments to achieving the Sustainable Development Goals (SDGs). In the United States (US), several students were often raped or sexually assaulted in places supposed to support safety^(1)^. In England, a survey reported that many students were victims of sexual violence on campuses. About 14% admitted to being victims of rapes, sexual assault, and unwanted touching^(2)^. In Indonesia, there were 82 cases of sexual violence reported to Komisi Nasional Anti Kekerasan terhadap Perempuan (National Commission on Violence Against Women) during 2021–2024^(3)^. However, only a small number of students experiencing sexual violence made a report. The lack of disclosure makes sexual violence on campus an iceberg phenomenon without showing the real numbers of cases.

Disclosure refers to the act of informing others about an experience of sexual violence through informal, formal,^(4)^ or online channels to obtain support, validation, justice, or awareness^(5)^. Several studies reported that sexual violence disclosure became a starting point to access legal assistance, medical, and psychological services^(6)^. Psychosocial and psychological assistance given to the victims of sexual violence is helpful for emotional recovery, trauma reduction, and social restoration^(7)^. Sexual violence disclosure can lead to negative consequences for the victims, such as the risk of retaliation, stigmatization, and other psychological attacks^(8)^.

The effect of sexual violence disclosure by the victims depends on social responses. Positive responses, including social support and trust, can facilitate recovery and empowerment among trauma survivors^(9,10)^. Negative responses can increase victims’ depression, shame, self-harm tendencies, and regret over disclosing their experiences^(9)^. Moreover, sexual violence disclosure, which is followed by no action, leads the victims to severe depression, a worse condition than disclosing the cases^(11)^. In higher institutions, disclosure brings a dilemma to the victims because of negative stigma, unequal power relations, and the lack of protection guarantees. These consequences make women, who in many cases of sexual violence are mostly the victims, reconsider the decision whether to disclose cases or keep silent.

Most women disclose sexual violence cases through informal methods. Several studies reported that most victims of sexual violence on campuses did not make a formal report. Almost 90% of the victims in Canada chose to remain silent, refraining from disclosing experiences and keeping cases confidential. Another study found that when a woman decides to report sexual violence cases, she prefers telling her friends or family members rather than the officials on campus^(12)^. A similar situation also happens in Indonesia. A study of six universities in Java found that only 33% of sexual violence cases on campus came to the desk of *Satuan Tugas Pencegahan dan Penanganan Kekerasan Seksual*/PPKS (a task force for sexual violence prevention and handling). Therefore, most cases result only in informal reporting, with a substantial proportion remaining unreported^(13)^. Despite the campus having issued regulations and units dealing with sexual violence on campus, the number of victims who dare to make a formal report is relatively low. This shows a gap between the formal framework and the sense of security given to the survivors.

Sexual violence is defined as any sexual act, unwanted comments or advances, or any other act directed at a person’s sexuality without consideration for the individual’s wishes or consent^(14)^. This includes sexual activity, with coercion or unequal power relations. Sexual violence can take the form of physical, non-physical, or gender-based sexual action. This term refers to the regulation of the Minister of Religious Affairs of 73/2022 on the prevention and handling of sexual violence in educational institutions. The regulation defines sexual violence in educational institutions into four categories, namely verbal, physical, non-physical, and digital sexual violence. Verbal sexual violence comprises sexually explicit or degrading remarks that humiliate or demean the victim. Meanwhile, non-physical sexual violence includes acts such as unwanted sexual advances, threats, lewd or lustful staring, and indecent exposure. Physical sexual violence entails touching, groping, attempted rape, rape, deception, and coercion of the victims. Digital sexual violence takes the form of sending, uploading, or downloading sexual content through digital devices in the form of sentences, images, audio, or video materials.

Sexual violence on campus is analyzed well using a combined framework of power relations, institutional betrayal, and feminist theory. The integration of the three perspectives explains the occurrence of sexual violence, the failure of campuses to protect members, and the perpetuation of injustice. According to Foucault, power is embedded in individuals and relationships, rules, discourses, and institutions. The power works through direct violence and norms, knowledge, and daily practices^(15)^. Sexuality represents a key domain through which power and knowledge influence reporting processes and determine the credibility of victims’ accounts^(16)^. Institutional betrayal is defined as failure in the form of inappropriate action or omission, affecting members^(17)^. This can take the form of the failure to prevent sexual violence, negative responses to the victims, and structures or policies given to protect the institution’s reputation^(17,18)^. From a feminist perspective, sexual violence is rooted in patriarchal structures that perpetuate unequal power relations between men and women, objectify women, and normalize violence within educational institutions. In feminist analysis, the silence shows how the rules and policies on campus allow the victims to keep silent to make a good image^(19)^.

Theoretical perspectives are particularly relevant to the Indonesian higher education context, where hierarchical campus structures and gendered social norms can shape the occurrence of sexual violence. Concerns about institutional reputation may also influence the occurrence of sexual violence and survivors’ willingness to report experiences. This study discussed the forms and prevalence of sexual violence on campuses, the barriers to making a formal report, and the psychological impact on the survivors. The integration of survey methods and in-depth interviews provided a comprehensive picture of how women decided to disclose cases amid psychosocial pressure.

## METHOD

This study used a mixed-methods approach with a sequential explanatory design, integrating quantitative and qualitative data in a step-by-step manner. In the first phase, quantitative data were collected through a survey of 472 respondents, including faculty leaders, lecturers, administrative staff, students, and other stakeholders from two campuses in Indonesia. Respondents were selected using a systematic random sampling to ensure representativeness of the target group. In the second phase, qualitative data were obtained through interviews with 47 informants to provide a more comprehensive explanation.

The instrument had been examined by some experts from psychology, sociology, and gender studies from some credible universities in Indonesia. In addition, the instrument was analyzed by some activists and officials from the National Commission on Violence Against Women. The quantitative instrument was designed to obtain information about campus perspectives and the understanding of sexual violence, to know responses to the issue, and to understand the prevalence of victims and perpetrators on campuses. A qualitative instrument was designed in the form of semi-structured interview guidance and an observation list. Quantitative and qualitative instruments were designed to understand the trend of sexual violence on campuses, responses to sexual violence, and the victims’ psychological condition after disclosure.

Data were analyzed using quantitative and qualitative approaches. Survey data were analyzed descriptively to report the prevalence of sexual violence on campus and the relationship between academic community understanding, institutional policies and responses, and experiences of victimization. Quantitative results were used to provide an overview of the conditions. Interview data were analyzed thematically through repeated reading of transcripts, data coding, categorization of codes, theme development, and review of the resulting themes ^(20)^. Thematic analysis is relevant to identify, analyze, and report the themes as well as explore victims’ experiences and perceptions of sexual violence on campuses.

### Ethical Aspects

This study has obtained ethical clearance from Badan Riset dan Inovasi Nasional (BRIN) under decree number 442/KE.01/SK/07/2023.

## RESULTS

### The Forms and Prevalence of Sexual Violence on Campuses

Based on the survey conducted on 472 respondents from two campuses, 12% experienced sexual violence in any form. From the survey on 220 respondents in Campus A, all forms of sexual violence occurred, except penetration. Therefore, verbal, physical, non-physical, or digital sexual violence was experienced or witnessed by the respondents of Campus A. At Campus B, 252 respondents reported that all forms of sexual violence occurred. This shows that the prevalence of sexual violence at Campus B was higher than that of Campus A.

The most common forms of sexual violence on the two campuses were verbal abuse, such as cat-calling, inappropriate utterances, or any obscene language. In addition, non-physical sexual violence, such as stalking, leering, and touching body parts, was reported by several respondents. Sending sexual materials through social media such as WhatsApp and Instagram was reported. Some lecturers used social media to access and save pictures of students without permission and sent sexual images.

Sexual violence happened in various places on campus, such as classrooms, halls, prayer rooms, corridors, meeting rooms for students and supervisors, even boarding houses and public spaces such as roads near campus. Therefore, sexual violence occurred in public and formal spaces as well as private and digital spaces. These sexual violence cases brought fear and inconvenience for the victims, as explained by an informant from Campus B.

*“I am a new student, and since the beginning, I have been stalked by my senior. He also sends me flirtatious messages on my Instagram and pursues me to have private chats on WhatsApp. He often secretly takes photos of me and peeks at me when I am in the classroom”*. (NA)

Sexual perpetrators on campus come from various backgrounds and professions, such as lecturers, senior students, or other persons around campus. In many cases, there is an unequal power relation between the perpetrators and the victims in which the power and position of the perpetrators are higher. In the case of thesis supervision, the students cannot refuse because the perpetrators were thesis supervisors. Furthermore, senior students or heads of certain organizations take advantage of emotional closeness and organizational structures to control victims in manipulative ways. The occurrence of all types of sexual violence on the two campuses reports that women continue to face safety risks within the educational environments. The perpetrators have strategic positions in academic or structural means that sexual violence has spread both horizontally and vertically within social structures on campus. These results reinforce that gender-based empowerment in preventing and responding to sexual violence in higher education is urgent.

This study analyzed four main variables, namely understanding of policies and campuses’ responses to sexual violence, as well as the prevalence of the victims and the perpetrators, to examine the relationship between the understanding of campus members. The data were collected from 472 respondents who participated, as presented in Table 1.

**Table 1:** The matrix of correlation between sexual violence policies understanding, responses of the campus, prevalence of the victims, and prevalence of the perpetrators – Indonesia, 2023.

Variable	Understanding sexual violence policies	Campuses’ responses to sexual violence	The prevalence of the victims	The prevalence of the perpetrators
Understanding sexual violence policies	1.000	0.320	-0.094	-0.056
Campuses’ responses to sexual violence	0.320	1.000	0.004	-0.010
The prevalence of the victims	-0.094	0.004	1.000	0.291
The prevalence of the perpetrators	-0.056	-0.010	0.291	1.000

Table 1 shows a fair positive correlation between understanding of sexual violence policies and campuses’ responses (r = 0.320), suggesting that greater policy awareness is associated with stronger institutional responses. A similar fair positive correlation was observed between the prevalence of victims and perpetrators (r = 0.291). The other correlation coefficients were negligible, showing minimal relationships among the remaining variables.

### Culture of Silence and Barriers to Reporting

This study asks an open-ended question to 472 respondents regarding the factors contributing to sexual violence to understand the interpretation in the campus environment. The answers range from personal moral factors, social relation patterns, social circle, to regulatory aspects and power relations on campus. Due to the wide variation and the use of diverse terms, all responses were categorized into the most relevant themes to enable a systematic interpretation of respondents’ perception patterns. Furthermore, the themes were analyzed and visualized using a word cloud to identify the terms that appeared most frequently and were most dominant in shaping college students’ understanding of the causes of sexual violence.

**Figure 1:**
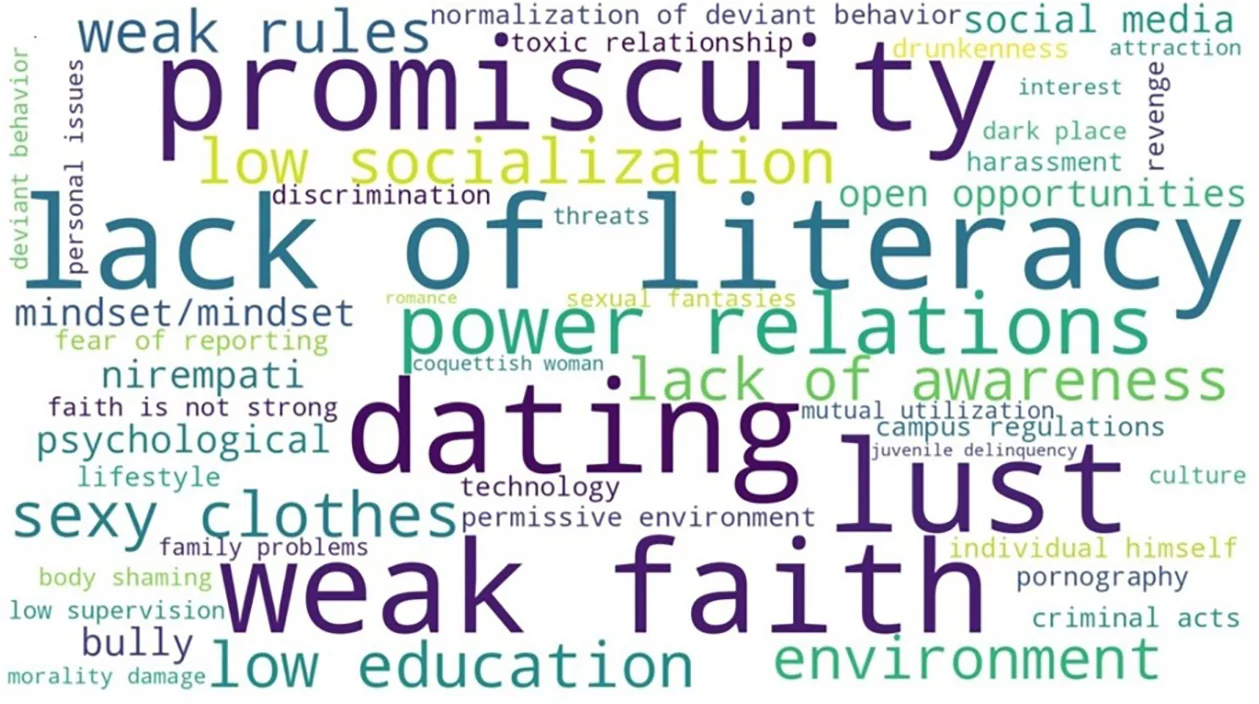
Visualization of the key terms that cause sexual violence on campus – Indonesia, 2023.

Figure 1 shows that the way respondents understand sexual violence on campus is strongly influenced by two perspectives, namely the moral and behavioral aspects of each individual. Terms such as “dating,” “promiscuity,” “lust,” “sexy clothing,” “weak faith,” and “lack of literacy” are reported as the most dominant responses, suggesting that sexual violence is often perceived as being primarily caused by individual desires or impulses, clothing choices, or the behaviors of victims and perpetrators. This result shows that many individuals continue to interpret sexual violence through a framework of everyday morality. Conversely, several other prominent terms are also considered, including “power relations,” “weak regulations,” “environment,” and “low awareness,” reflecting a broader understanding of sexual violence as being influenced by structural and contextual factors. Some respondents recognize that sexual violence is not solely an individual problem. Several structural factors may contribute to the occurrence, including senior–junior power relations, lecturer–student relationships, inadequate campus regulations, and limited understanding of consent and sexual violence. Structural terms appeared less prominently than morality-based terms, such as “lust” and “sexy clothing.” This shows that perceptions of sexual violence within the campus environment remain situated between two perspectives. First, some individuals continue to perceive the concept primarily as an individual moral failure. Second, some respondents acknowledge that cultural norms, power relations, and institutional systems create conditions enabling the occurrence of sexual violence. The development of terms such as “fear of reporting,” “campus regulations,” and “psychological” reflects an awareness that victims may face significant barriers and challenging circumstances when reporting cases.

**Figure 2:**
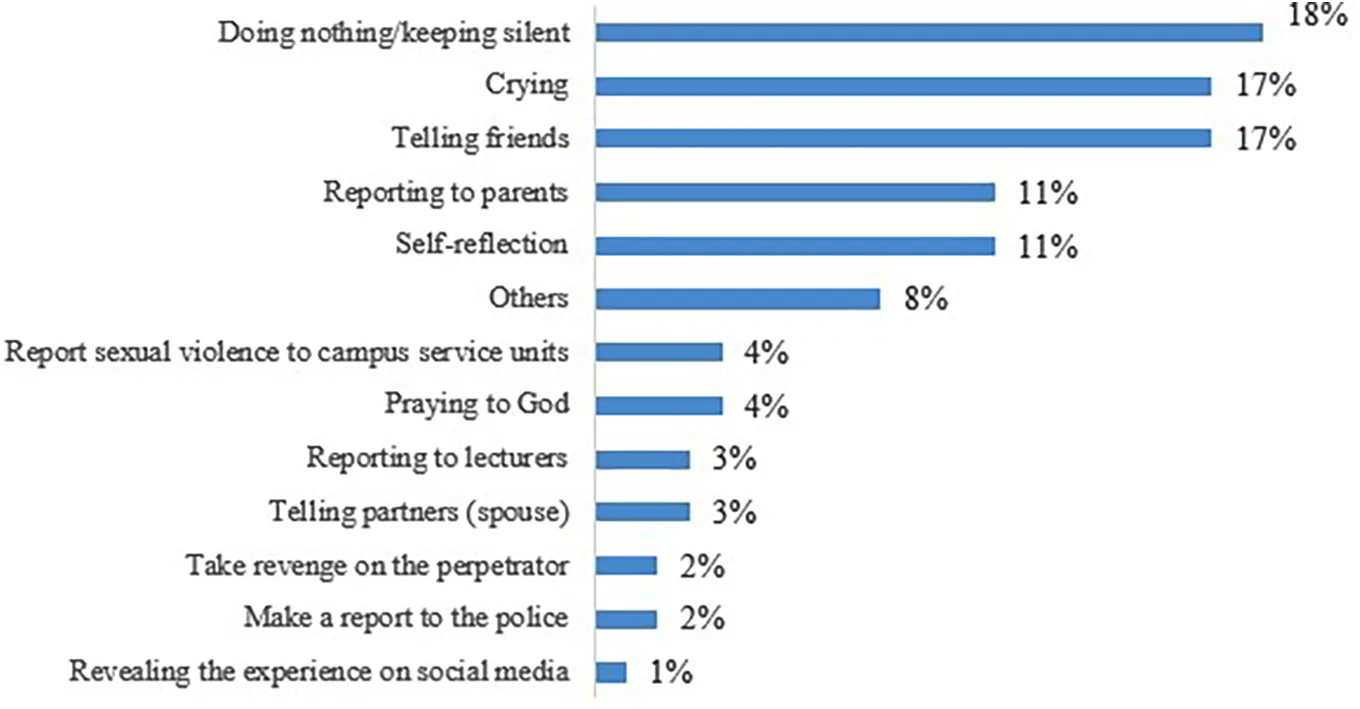
Various responses given by respondents experiencing sexual violence – Indonesia, 2023.

The campus community’s understanding of sexual violence also appears to shape victims’ responses to experiences of sexual violence. Even though the prevalence of sexual violence is relatively high, the victims tend to remain silent. Approximately 12% of the respondents claiming to experience sexual violence were further asked, “what they did related to the sexual violence they experienced”. Among the 114 responses, “doing nothing or keeping silent” was the most frequently reported answer, selected by 18% of the respondents. The percentage of various answers to this advanced question is presented in Figure 2.

The results were supported by data from interviews with some officials of service units for sexual violence at the two campuses. In Campus A, only four cases were reported to the service unit, while the informants handled a minimum of eight cases. There were twelve cases in Campus B officially handled by the service unit from 2022 to 2023. The cases comprised students, lecturers, staff, and individuals from outside the campus. These results show the phenomenon of a lack report of sexual violence cases. Most victims of Campus B did not make a formal report to the service unit. The incidents were told to close friends or senior ones, not to the service unit. This is in line with students’ perceptions, where 30% of the respondents were unsure about the ability of the campus to handle cases. In this context, 23% and 33% stated that the campus did not have reporting and support systems, respectively. Therefore, lower trust in the campus system exacerbates the level of silence surrounding sexual violence cases.

Some main barriers to making a report are fear, trauma, negative social stigma, and ignorance about the support mechanisms. In one case, the victims did not know that the campus had a service unit for sexual violence. In another case, a report was not made because of shame and fear of making the cases worse. Furthermore, the victim considered the case was not worth reporting because the incident occurred outside the campus and the perpetrator was not a student. The fear that reporting would worsen personal and academic situations is another reason for sexual violence victims to keep silent. Fear was even greater when the perpetrator held a strategic position. The report would lead to negative consequences for the academic journey, specifically when the perpetrator was a structural member of the campus. An activist stated the following.

*“Actually, there was a case at the faculty. The perpetrators were an official and the head of a study program. The student, who was the victim, was afraid that her thesis process would become more difficult, and that is another obstacle. Many sexual violence cases were kept secret, specifically when the perpetrators were university officials”. (DM)*

Power relations are another factor affecting victims from making a report. In some cases, the perpetrators are thesis supervisors, senior students, or members of a certain organization. Victims are in a subordinate position and lack control over the reporting process and outcomes. This situation places victims in a difficult social and academic position when the incident is reported. The challenge is further compounded when victims receive no support from family members. The responses received were characterized by distrust and ignorance. The culture of silence, formed from power abuse, stigmatization, and distrust of the institution, makes campuses unsafe places, specifically for women and other vulnerable groups.

### Psychological Impacts on the Victims

Sexual violence results in severe and long-lasting psychological consequences for the survivors. At Campus A, a survivor who was sexually abused by the “older brother” felt she was “no longer pure” and worried about the future. She cried and reported feeling that her dignity and moral integrity had been violated. An extreme case of sexual violence also occurred in Campus A, where a female student was repeatedly sexually abused by the supervisor. Due to sustained psychological pressure, she developed severe depression, engaged in self-harm by cutting her arms in a certain pattern, and made several suicide attempts. The severely destabilized mental condition rendered her unable to feel physical pain when the wounds were treated, suggesting that the trauma had surpassed the threshold of physical perception.

In Campus B, sexual violence led to significant psychological impacts on the victims. The officials of the service unit who treated and helped victims for recovery reported that most individuals got depressed and made suicide. Therefore, intensive psychological support is required for an effective recovery. The officials of the service unit for sexual violence also gave primary focus on addressing deep psychological recovery from the trauma.

Another impact of sexual violence on the victims is decreased academic achievement, as experienced by a female student of Campus B. There was no motivation to continue the thesis supervision process following the reluctance to meet the supervisor, who had sexually harassed her. Based on the interview with the campus health professional, the female student was reported to suffer from acute stress, persistent nausea, and psychosomatic symptoms. Physical and medical intervention would be inadequate since the primary need was psychological rehabilitation. The underlying cause of the condition was psychological trauma due to sexual harassment endured.

Sexual violence survivors tend to be isolated from social life. These individuals tend to withdraw from academic activities on campus, avoid public spaces, and feel uncomfortable interacting with the opposite sex. In milder cases, some survivors felt more comfortable when close friends accompany activities on campus. This traumatic experience leads to a negative sense of security as narrated by an informant:

*“The survivor is sometimes reluctant to attend a meeting on campus. We made an appointment to meet in a certain place outside campus, such as a cafe or another place. Because they have traumatic memories of campus, they avoid it”. (AF)*

After deciding to report the cases, the survivors at the two campuses experienced more complex psychological consequences, from deep trauma to feeling helpless because of inadequate responses. At campus B, the survivors remained fearful because the perpetrators remained on campus while reports had not been addressed. Even some survivors completely avoided going to campus. At Campus A, some survivors experienced secondary victimization, dealing with the initial assault, social stigma, and the lack of systemic support. Some female survivors felt “no longer pure” and persistently anxious about the future. Others continued to suffer from harassment from faculty members despite having filed formal complaints.

These cases show a constant pattern of institutional failure in preventing and handling sexual violence on campuses. Victims who are willing to report cases of sexual violence on campus often experience powerlessness and become trapped in a state of fear and uncertainty.

## DISCUSSION

Sexual violence on campus deals with power imbalances and inadequate institutional support for the prevention, as shown in a survey of 472 respondents and an in-depth interview with 47 individuals. Foucault’s theory of Power Relation explains how hierarchical structures such as seniority, religious authority, and dynamic relations between student and lecturer create a space for exploitation where perpetrators use dominant positions to normalize sexual violence. In this context, power relations show the relational, scattered, and productive nature^(21)^ that allows sexual violence to occur on campus. The violence is due to individual domination, authority networks, norms, and social interaction in the academic environment. Foucault stated that power was not only repressive but also productive^(22)^. In this framework, campuses = produce a discourse that classifies behaviors into “appropriate” and “inappropriate”. This categorization leads individuals to blame survivors because of the violation of the norms. Studies about the culture of shame show that sexual violence cases are often silenced for institutional reputation, while the perpetrators are saved by powerful networks^(23)^.

The high vulnerability of sexual violence on campus cannot be separated from the institutional conditions and the implementation of policies that are still weak. Even though some campuses have established integrated service units as part of prevention and handling, the existence of the unit in practice has not been fully supported by authorities. In one case, the service unit experienced limitations due to the absence of financial support. This affected the work of handling, assisting victims, and educating the academic community. The situation suggests that the existence of formal policies is insufficient unless accompanied by genuine institutional commitment. Some lecturers’ perception of verbal sexual violence as a “normal” occurrence reflects the continued normalization of cases. This condition presents additional challenges for the service unit since the responsibilities include handling cases of sexual violence and addressing misconceptions within the campus community. Therefore, the effectiveness of campus policies is undermined, while students’ trust in the institution’s commitment to addressing sexual violence is also decreased.

The slow responses of the campuses to sexual violence cases increase the impact on the survivors. This condition reflects what Holland and Cortina called “institutional betrayal” when an institution fails to fulfil the function as a protector^(24)^. Campuses often prioritize reputation by concealing sexual violence cases or protecting the perpetrators. These practices are consistent with the “patriarchal bargain” concept^(25)^, where females are forced to negotiate with a disadvantaged system. In another study^(26)^, the reluctance to submit formal reports due to fear of being blamed, dismissed, or accused of damaging the institution’s reputation constitutes evidence of the institutionalization of patriarchal culture within campus policies.

This study empirically showed the failure of campuses to establish trust among survivors (Figure 2) since only 4% reported sexual violence experiences to the service unit. Meanwhile, informal disclosure was often conducted through speaking to the parents, friends, and spouse. This result is consistent with previous studies, where survivors tend to prefer informal disclosure over formal reporting to the police, campus-based service units, or other authorized institutions providing services^(27)^. The decision may be influenced by several factors, including fear of victim-blaming, perceptions of overreaction, social stigma, close relationships with perpetrators, and negative experiences reported by other survivors who previously disclosed cases^(27)^. Informal disclosure is frequently conducted through gradual and selective conversation^(28)^. This study also found that the different forms of sexual violence had not been sufficiently socialized among the campus community.

Feminists extend Foucault’s analysis by emphasizing that control over female bodies is the core of patriarchy^(29)^. In the context of the campus environment, control over the female body is often legitimized by gender-biased moral discourse. This result was shown by the present study, where several factors perceived as contributing to sexual violence included “sexy clothes,” “body shaming,” and references to “flirtatious women.” Conversely, perpetrators’ actions were justified through references to “dating,” “promiscuity,” “weak faith,” “lust,” and “lifestyle.” These results were relevant to other previous studies, where sexual violence was often rationalized through the idea that men were inherently dominant, aggressive, and unable to control sexual desires^(30)^.

Based on the preceding description, this study reports another important aspect, namely the growing willingness among women to disclose experiences of sexual violence. The courage did not appear spontaneously but was supported by peer networks, student organizations, and service units for sexual violence. This collective support strengthened the position in dealing with trauma and provided a safe space to report cases without worrying about stigma. However, the lack of adequate institutional support left service unit volunteers feeling isolated in efforts to address sexual violence cases. In Campus B, the process of drafting Standard Operating Procedures (SOP) for prevention took a long time due to the absence of adequate understanding of the indicators in the form of verbal abuse. Another systematic study reports that campus officials or structures obstructing prevention programs are a kind of institutional betrayal^(18)^.

## CONCLUSION

In conclusion, sexual violence on campuses took several forms, ranging from verbal, physical, and digital to coercion, with high prevalence. The trauma leads to serious psychological consequences for the survivors, specifically females who decided to speak up about the experiences. Some of the psychological consequences are prolonged anxiety, loss of self-esteem, severe depression, and suicide attempts. Psychosocial assistance provided by the service units for sexual violence prevention at Campuses A and B was proven significantly crucial to mental health. However, religious and legal assistance provided by the campuses was insufficient. This study also confirmed that power dynamics, slow responses of the institutions, and social stigma collectively shaped more complex psychological problems. The number of females courage to speak up about sexual violence experiences has increased. The courage is supported by peer networks, student organizations, and the service units of the campuses.

The implications show that campuses need to strengthen the sexual violence protection and handling system more thoroughly, focusing on the reporting mechanism and psychosocial support. Therefore, higher education institutions need to provide more comprehensive services, such as counseling, legal aid, and inclusive spiritual support, allowing the recovery process to run more safely and sustainably. Internal regulation strengthening and implementing clear and firm punishment becomes an important action to prevent the neglect of perpetrators. The results affirm the need for integration of gender equality education, relational ethics, and sensitivity training in the learning process. The issue is important to consider given the strong moralistic perspectives and the normalization of sexual violence within the campus community’s understanding. In addition, cross-sectoral collaboration among campuses, service units, companion agencies, and civil society organizations needs to be strengthened to develop a comprehensive prevention and recovery system.

This study was conducted only at two state universities, restricting the broader generalization to the national education context. The domination of quantitative descriptive analysis reduces the capacity to make causal inferences about the longitudinal recovery of survivors. Another limitation was that the informants were selected from the individuals who disclosed cases. Therefore, this study neglected the perspectives of the survivors who decided to keep silent because of social stigma, institutional barriers, and other reasons.

Future study could expand institutional scope by including private universities and applying longitudinal analysis to systematically trace survivors’ recovery. The used methods should be integrated with an inferential approach to obtain a deep understanding of institutional responses, survivors’ coping strategies, and academic culture.

## DATA AVAILABILITY

The participants did not give written consent for the data to be shared publicly. Therefore, the supporting data are unavailable due to the sensitive nature of the study.
