# Supplementary material for: Breaking the silence: examining campus sexual violence and the psychological effects on women survivors
Source: Rev Esc Enferm USP. 2026 Aug 24;60:e20260155. doi: 10.1590/1980-220X-REEUSP-2026-0155en (PMC13506164; doi:10.1590/1980-220X-REEUSP-2026-0155en)
Supplement: Translation

# Translation: Rompiendo el silencio: examinando la violencia sexual en los campus universitarios y sus efectos psicológicos en las mujeres sobrevivientes

**DOI:** 10.1590/1980-220X-REEUSP-2026-0155es

**Authors:** Umi Muzayanah, Siti Muawanah, Nur Laili Noviani, Arif Gunawan Santoso, Dwi Martiningsih

**Affiliations:** 1 National Research and Innovation Agency, Research Organization for Social Sciences and Humanities, Research Center for Religion and Belief, Jakarta, Indonesia.; 2 Universitas Islam Negeri Salatiga, Doctoral Program, Postgraduate School, Islamic Education, Salatiga, Indonesia.

**Keywords:** Universidades, Delitos Sexuales, Sobrevivientes Mujeres, Revelación.

## Abstract

**Objetivo:**

Explorar la violencia sexual en la educación superior en Indonesia y su impacto psicológico en las mujeres que desean denunciarla.

**Método:**

Este estudio de métodos mixtos combinó una encuesta en línea a 472 participantes, incluyendo estudiantes, docentes, funcionarios y personal de dos universidades, con entrevistas de seguimiento para enriquecer y validar los hallazgos de la encuesta.

**Resultados:**

Los resultados revelan que la violencia sexual se manifiesta de diversas formas en el campus. El 12% de los participantes reportaron haber sido víctimas, mientras que el 18% prefirieron no revelar sus experiencias. Este estudio también identificó algunos factores que contribuyen a la violencia sexual en los campus, como la presión de grupo, el abuso de poder, la falta de valores morales y la capacidad para cometer actos de violencia sexual. Algunas víctimas reportaron haber enfrentado problemas psicológicos significativos, como trauma, miedo, depresión, deseos de autolesionarse y una disminución en su rendimiento académico.

**Conclusión:**

El estudio destaca la necesidad de que los campus implementen intervenciones integrales para las sobrevivientes de violencia sexual, con el fin de crear un entorno académico más seguro y receptivo.

## INTRODUCCIÓN

La creciente prevalencia de la violencia sexual en los campus es profundamente preocupante y refleja una contradicción con los compromisos globales para alcanzar los Objetivos de Desarrollo Sostenible (ODS). En Estados Unidos (EE. UU.), varias estudiantes fueron violadas o agredidas sexualmente con frecuencia en lugares que se supone deben garantizar su seguridad^(1)^. En Inglaterra, una encuesta reveló que muchas estudiantes fueron víctimas de violencia sexual en los campus. Alrededor del 14% admitió haber sido víctima de violaciones, agresiones sexuales y tocamientos no deseados^(2)^. En Indonesia, se reportaron 82 casos de violencia sexual ante la Komisi Nasional Anti Kekerasan terhadap Perempuan (Comisión Nacional contra la Violencia hacia las Mujeres) durante el período 2021-2024^(3)^. Sin embargo, solo un pequeño número de estudiantes que sufrieron violencia sexual presentó una denuncia. La falta de denuncia convierte a la violencia sexual en los campus en un fenómeno tipo “iceberg”, sin que se conozcan las cifras reales de los casos.

La denuncia se refiere al acto de informar a otros sobre una experiencia de violencia sexual a través de canales informales, formales^(4)^ o en línea para obtener apoyo, validación, justicia o concientización^(5)^. Varios estudios indicaron que la denuncia de la violencia sexual se convirtió en un punto de partida para acceder a asistencia legal, servicios médicos y psicológicos^(6)^. La asistencia psicosocial y psicológica brindada a las víctimas de violencia sexual es útil para la recuperación emocional, la reducción del trauma y la reintegración social^(7)^. La denuncia de la violencia sexual puede acarrear consecuencias negativas para las víctimas, como el riesgo de represalias, estigmatización y otros ataques psicológicos^(8)^.

El efecto de la denuncia de la violencia sexual por parte de las víctimas depende de las respuestas sociales. Las respuestas positivas, que incluyen el apoyo social y la confianza, pueden facilitar la recuperación y el empoderamiento de quienes han sobrevivido al trauma^(9,10)^. Las respuestas negativas pueden aumentar la depresión, la vergüenza, las tendencias a la autolesión y el arrepentimiento de las víctimas por haber dado a conocer sus experiencias^(9)^. Además, la denuncia de la violencia sexual, cuando no va seguida de ninguna acción, lleva a las víctimas a una depresión grave, una situación peor que la de denunciar los casos^(11)^. En las instituciones de educación superior, la denuncia plantea un dilema a las víctimas debido al estigma negativo, las relaciones de poder desiguales y la falta de garantías de protección. Estas consecuencias hacen que las mujeres, que en muchos casos de violencia sexual son en su mayoría las víctimas, se replanteen la decisión de denunciar los casos o guardar silencio.

La mayoría de las mujeres denuncian los casos de violencia sexual a través de métodos informales. Varios estudios indicaron que la mayoría de las víctimas de violencia sexual en los campus universitarios no presentaron una denuncia formal. Casi el 90% de las víctimas en Canadá optaron por guardar silencio, absteniéndose de revelar sus experiencias y manteniendo la confidencialidad de los casos. Otro estudio reveló que, cuando una mujer decide denunciar un caso de violencia sexual, prefiere contárselo a sus amigos o familiares en lugar de a las autoridades del campus^(12)^. Una situación similar también ocurre en Indonesia. Un estudio realizado en seis universidades de Java reveló que solo el 33% de los casos de violencia sexual en el campus llegaron a la mesa de la *Satuan Tugas Pencegahan dan Penanganan Kekerasan Seksual*/PPKS (un grupo de trabajo para la prevención y el manejo de la violencia sexual). Por lo tanto, la mayoría de los casos solo dan lugar a denuncias informales, y una proporción considerable queda sin denunciar^(13)^. A pesar de que el campus cuenta con normativas y unidades encargadas de atender la violencia sexual en el campus, el número de víctimas que se atreven a presentar una denuncia formal es relativamente bajo. Esto pone de manifiesto una brecha entre el marco formal y la sensación de seguridad que se brinda a las sobrevivientes.

La violencia sexual se define como cualquier acto sexual, comentarios o insinuaciones no deseados, o cualquier otro acto dirigido a la sexualidad de una persona sin tener en cuenta sus deseos o su consentimiento^(14)^. Esto incluye la actividad sexual con coacción o en el marco de relaciones de poder desiguales. La violencia sexual puede manifestarse como acción sexual física, no física o basada en el género. Este término se refiere a la norma del Ministro de Asuntos Religiosos n.º 73/2022 sobre la prevención y el manejo de la violencia sexual en instituciones educativas. La norma define la violencia sexual en instituciones educativas en cuatro categorías: violencia sexual verbal, física, no física y digital. La violencia sexual verbal comprende comentarios sexualmente explícitos o degradantes que humillan o menosprecian a la víctima. Por su parte, la violencia sexual no física incluye actos como insinuaciones sexuales no deseadas, amenazas, miradas lascivas o lujuriosas y exhibicionismo. La violencia sexual física implica tocar, manosear, intento de violación, violación, engaño y coacción hacia las víctimas. La violencia sexual digital consiste en enviar, subir o descargar contenido sexual a través de dispositivos digitales en forma de textos, imágenes, audio o videos.

La violencia sexual en los campus se analiza adecuadamente mediante un marco combinado de relaciones de poder, traición institucional y teoría feminista. La integración de estas tres perspectivas explica la ocurrencia de la violencia sexual, el fracaso de los campus para proteger a sus miembros y la perpetuación de la injusticia. Según Foucault, el poder está arraigado en los individuos y las relaciones, las reglas, los discursos y las instituciones. El poder opera a través de la violencia directa y de las normas, el conocimiento y las prácticas cotidianas^(15)^. La sexualidad representa un ámbito clave a través del cual el poder y el conocimiento influyen en los procesos de denuncia y determinan la credibilidad de los relatos de las víctimas^(16)^. La traición institucional se define como un incumplimiento que toma la forma de una acción inapropiada u omisión, y que afecta a los miembros^(17)^. Esto puede manifestarse en la incapacidad de prevenir la violencia sexual, en respuestas negativas hacia las víctimas y en estructuras o políticas diseñadas para proteger la reputación de la institución^(17,18)^. Desde una perspectiva feminista, la violencia sexual tiene sus raíces en estructuras patriarcales que perpetúan relaciones de poder desiguales entre hombres y mujeres, cosifican a las mujeres y normalizan la violencia dentro de las instituciones educativas. En el análisis feminista, el silencio revela cómo las reglas y políticas en el campus permiten que las víctimas guarden silencio para mantener una buena imagen^(19)^.

Las perspectivas teóricas son particularmente relevantes para el contexto de la educación superior en Indonesia, donde las estructuras jerárquicas del campus y las normas sociales de género pueden influir en la ocurrencia de la violencia sexual. La preocupación por la reputación institucional también puede influir en la ocurrencia de la violencia sexual y en la disposición de las sobrevivientes a denunciar sus experiencias. Este estudio analizó las formas y la prevalencia de la violencia sexual en los campus, las barreras para presentar una denuncia formal y el impacto psicológico en las sobrevivientes. La integración de métodos de encuesta y entrevistas en profundidad proporcionó un panorama integral de cómo las mujeres decidieron dar a conocer los casos en medio de la presión psicosocial.

## MÉTODO

Este estudio utilizó un enfoque de métodos mixtos con un diseño explicativo secuencial, integrando datos cuantitativos y cualitativos de manera gradual. En la primera fase, se recolectaron datos cuantitativos mediante una encuesta a 472 participantes, entre los que se incluían líderes del cuerpo docente, profesores, personal administrativo, estudiantes y otras partes interesadas de dos campus en Indonesia. Los participantes fueron seleccionados mediante un muestreo aleatorio sistemático para garantizar la representatividad del grupo objetivo. En la segunda fase, se obtuvieron datos cualitativos mediante entrevistas a 47 informantes para proporcionar una explicación más exhaustiva.

El instrumento había sido revisado por expertos en psicología, sociología y estudios de género de varias universidades reconocidas de Indonesia. Además, el instrumento fue analizado por activistas y funcionarios de la Comisión Nacional contra la Violencia hacia las Mujeres. El instrumento cuantitativo se diseñó para obtener información sobre las perspectivas de los campus y la comprensión de la violencia sexual, conocer las respuestas ante este problema y comprender la prevalencia de víctimas y agresores en los campus. Se diseñó un instrumento cualitativo en forma de guía para entrevistas semiestructuradas y una lista de observación. Los instrumentos cuantitativos y cualitativos se diseñaron para comprender la tendencia de la violencia sexual en los campus, las respuestas ante la violencia sexual y el estado psicológico de las víctimas tras la denuncia.

Los datos se analizaron utilizando enfoques cuantitativos y cualitativos. Los datos de la encuesta se analizaron de manera descriptiva para informar sobre la prevalencia de la violencia sexual en el campus y la relación entre la comprensión de la comunidad académica, las políticas y respuestas institucionales, y las experiencias de victimización. Los resultados cuantitativos se utilizaron para ofrecer una visión general de la situación. Los datos de las entrevistas se analizaron temáticamente mediante la lectura repetida de las transcripciones, la codificación de los datos, la categorización de los códigos, el desarrollo de temas y la revisión de los temas resultantes^(20)^. El análisis temático es relevante para identificar, analizar y dar a conocer los temas, así como para explorar las experiencias y percepciones de las víctimas respecto a la violencia sexual en los campus.

### Aspectos Éticos

Este estudio ha obtenido la autorización ética de la Badan Riset dan Inovasi Nasional (BRIN) en virtud del decreto número 442/KE,01/SK/07/2023.

## RESULTADOS

### Las Formas y la Prevalencia de la Violencia Sexual en los Campus

Según la encuesta realizada a 472 participantes de dos campus, el 12% sufrió violencia sexual en alguna de sus formas. De la encuesta a 220 participantes del Campus A, se registraron todas las formas de violencia sexual, excepto la penetración. Por lo tanto, los participantes del Campus A sufrieron o presenciaron violencia sexual verbal, física, no física o digital. En el Campus B, 252 participantes informaron que se produjeron todas las formas de violencia sexual. Esto demuestra que la prevalencia de la violencia sexual en el Campus B fue mayor que en el Campus A.

Las formas más comunes de violencia sexual en los dos campus fueron el abuso verbal, como piropos, comentarios inapropiados o cualquier lenguaje obsceno. Además, varios encuestados reportaron violencia sexual no física, como acoso, miradas lascivas y tocamientos en partes del cuerpo. También se reportó el envío de material sexual a través de redes sociales como WhatsApp e Instagram. Algunos profesores utilizaban las redes sociales para acceder y guardar fotos de los estudiantes sin permiso, y enviaban imágenes de carácter sexual.

La violencia sexual ocurrió en diversos lugares del campus, como aulas, salones, salas de oración, pasillos, salas de reuniones para estudiantes y supervisores, incluso en residencias estudiantiles y espacios públicos como las calles cercanas al campus. Por lo tanto, la violencia sexual ocurrió tanto en espacios públicos y formales como en espacios privados y digitales. Estos casos de violencia sexual generaron miedo e incomodidad en las víctimas, tal como lo explicó una informante del Campus B.

*“Soy una estudiante nueva y, desde el principio, un estudiante de cursos superiores me ha estado acosando. También me envía mensajes coquetos por Instagram y me insiste para que tengamos pláticas privadas por WhatsApp. A menudo me toma fotos a escondidas y me espía cuando estoy en el salón de clases”.* (NA)

Los agresores sexuales en el campus provienen de diversos orígenes y profesiones, como profesores, estudiantes de cursos superiores u otras personas del entorno del campus. En muchos casos, existe una relación de poder desigual entre los agresores y las víctimas, en la que el poder y la posición de los agresores son superiores. En el caso de la supervisión de tesis, las estudiantes no pueden negarse porque los agresores son sus supervisores de tesis. Además, los estudiantes de cursos superiores o los líderes de ciertas organizaciones se aprovechan de la cercanía emocional y las estructuras organizativas para controlar a las víctimas de manera manipuladora. La presencia de todo tipo de violencia sexual en los dos campus indica que las mujeres siguen enfrentando riesgos de seguridad dentro de los entornos educativos. Los agresores ocupan puestos estratégicos en el ámbito académico o estructural, lo que significa que la violencia sexual se ha extendido tanto horizontal como verticalmente dentro de las estructuras sociales del campus. Estos resultados refuerzan la urgencia de un empoderamiento con perspectiva de género para prevenir y responder a la violencia sexual en la educación superior.

Este estudio analizó cuatro variables principales, a saber, la comprensión de las políticas y las respuestas de los campus ante la violencia sexual, así como la prevalencia de las víctimas y los agresores, para examinar la relación entre la comprensión de los miembros del campus. Los datos se recolectaron de 472 encuestados que participaron, tal como se presenta en la Tabla 1.

**Tabla 1:** Matriz de correlación entre la comprensión de las políticas sobre violencia sexual, las respuestas del campus, la prevalencia de las víctimas y la prevalencia de los agresores – Indonesia, 2023.

Variable	Comprender las políticas sobre violencia sexual	Respuestas de las universidades ante la violencia sexual	Prevalencia de las víctimas	Prevalencia de los agresores
Comprender las políticas sobre violencia sexual	1,000	0,320	-0,094	-0,056
Respuestas de las universidades ante la violencia sexual	0,320	1,000	0,004	-0,010
Prevalencia de las víctimas	-0,094	0,004	1,000	0,291
Prevalencia de los agresores	-0,056	-0,010	0,291	1,000

La Tabla 1 muestra una correlación positiva moderada entre la comprensión de las políticas sobre violencia sexual y las respuestas de los campus (r = 0,320), lo que sugiere que un mayor conocimiento de las políticas se asocia con respuestas institucionales más sólidas. Se observó una correlación positiva moderada similar entre la prevalencia de víctimas y agresores (r = 0,291). Los demás coeficientes de correlación fueron insignificantes, lo que indica relaciones mínimas entre las variables restantes.

### Cultura del Silencio y Barreras para Denunciar

Este estudio plantea una pregunta abierta a 472 encuestados sobre los factores que contribuyen a la violencia sexual, con el fin de comprender su interpretación en el entorno universitario. Las respuestas abarcan desde factores morales personales, patrones de relaciones sociales y el círculo social, hasta aspectos normativos y relaciones de poder en el campus. Debido a la amplia variación y al uso de diversos términos, todas las respuestas se clasificaron en los temas más relevantes para permitir una interpretación sistemática de los patrones de percepción de los encuestados. Además, los temas se analizaron y visualizaron mediante una nube de palabras para identificar los términos que aparecían con mayor frecuencia y que eran más determinantes en la forma en que los estudiantes universitarios entendían las causas de la violencia sexual.

La Figura 1 muestra que la forma en que los encuestados entienden la violencia sexual en el campus está fuertemente influenciada por dos perspectivas: los aspectos morales y de comportamiento de cada individuo. Términos como “citas”, “promiscuidad”, “lujuria”, “ropa sexy”, “fe débil” y “falta de educación” figuran como las respuestas más frecuentes, lo que sugiere que la violencia sexual suele percibirse como algo causado principalmente por los deseos o impulsos individuales, las elecciones de vestimenta o los comportamientos de las víctimas y los agresores. Este resultado muestra que muchas personas siguen interpretando la violencia sexual a través de un marco de moralidad cotidiana. Por el contrario, también se consideraron otros términos destacados, como “relaciones de poder”, “regulaciones débiles”, “entorno” y “baja concientización”, lo que refleja una comprensión más amplia de la violencia sexual como un fenómeno influenciado por factores estructurales y contextuales. Algunos encuestados reconocen que la violencia sexual no es únicamente un problema individual. Varios factores estructurales pueden contribuir a su ocurrencia, entre ellos las relaciones de poder entre estudiantes de cursos superiores e inferiores, las relaciones entre profesores y estudiantes, las regulaciones inadecuadas en el campus y la comprensión limitada del consentimiento y la violencia sexual. Los términos estructurales aparecieron con menos frecuencia que los basados en la moralidad, como “lujuria” y “ropa sexy”. Esto demuestra que las percepciones sobre la violencia sexual en el entorno universitario siguen situándose entre dos perspectivas. En primer lugar, algunas personas siguen percibiendo el concepto principalmente como un fracaso moral individual. En segundo lugar, algunos encuestados reconocen que las normas culturales, las relaciones de poder y los sistemas institucionales crean condiciones que facilitan la ocurrencia de la violencia sexual. La aparición de términos como “miedo a denunciar”, “normas del campus” y “psicológico” refleja la conciencia de que las víctimas pueden enfrentar barreras significativas y circunstancias difíciles al denunciar los casos.

**Figura 1:** Visualización de los términos clave (en inglés) que dan lugar a la violencia sexual en el campus —Indonesia, 2023.

La forma en que la comunidad universitaria entiende la violencia sexual también parece influir en las respuestas de las víctimas ante las experiencias de violencia sexual. Aunque la prevalencia de la violencia sexual es relativamente alta, las víctimas tienden a guardar silencio. A aproximadamente el 12% de los encuestados que afirmaron haber sufrido violencia sexual se les preguntó además “qué hicieron al respecto de la violencia sexual que sufrieron”. Entre las 114 respuestas, “no hacer nada o guardar silencio” fue la respuesta más frecuente, seleccionada por el 18% de los encuestados. El porcentaje de las distintas respuestas a esta pregunta complementaria se presenta en la Figura 2.

**Figura 2:** Diversas respuestas dadas por las personas encuestadas que sufrieron violencia sexual —Indonesia, 2023.

Los resultados se vieron respaldados por datos de entrevistas con algunos funcionarios de las unidades de atención a la violencia sexual en los dos campus. En el Campus A, solo se denunciaron cuatro casos ante la unidad de atención, mientras que los informantes manejaron al menos ocho casos. En el Campus B hubo doce casos atendidos oficialmente por la unidad de atención entre 2022 y 2023. Los casos involucraron a estudiantes, profesores, personal y personas ajenas al campus. Estos resultados muestran el fenómeno de la falta de denuncia de casos de violencia sexual. La mayoría de las víctimas del Campus B no presentaron una denuncia formal ante la unidad de atención. Los incidentes se contaron a amigos cercanos o a personas de mayor edad, no a la unidad de atención. Esto concuerda con las percepciones de los estudiantes, ya que el 30% de los encuestados no estaba seguro de la capacidad del campus para manejar los casos. En este contexto, el 23% y el 33% afirmaron que el campus no contaba con sistemas de denuncia y de apoyo, respectivamente. Por lo tanto, la menor confianza en el sistema del campus agrava el nivel de silencio que rodea a los casos de violencia sexual.

Algunas de las principales barreras para presentar una denuncia son el miedo, el trauma, el estigma social negativo y el desconocimiento de los mecanismos de apoyo. En un caso, las víctimas no sabían que el campus contaba con una unidad de atención para la violencia sexual. En otro caso, no se presentó una denuncia debido a la vergüenza y al miedo a que la situación empeorara. Además, la víctima consideró que no valía la pena denunciar el caso porque el incidente ocurrió fuera del campus y el agresor no era estudiante. El temor a que la denuncia empeore la situación personal y académica es otra razón por la que las víctimas de violencia sexual guardan silencio. El miedo era aún mayor cuando el agresor ocupaba un puesto estratégico. La denuncia traería consecuencias negativas para la trayectoria académica, especialmente cuando el agresor era un miembro de la estructura administrativa del campus. Una activista declaró lo siguiente:

*“De hecho, hubo un caso en la facultad. Los agresores eran un funcionario y el director de un programa de estudios. La estudiante, que era la víctima, temía que el proceso de su tesis se volviera más difícil, y ese es otro obstáculo. Muchos casos de violencia sexual se mantuvieron en secreto, especialmente cuando los agresores eran funcionarios de la universidad”.* (DM)

Las relaciones de poder son otro factor que influye en que las víctimas no presenten una denuncia. En algunos casos, los agresores son directores de tesis, estudiantes de cursos superiores o miembros de cierta organización. Las víctimas se encuentran en una posición subordinada y carecen de control sobre el proceso de denuncia y sus resultados. Esta situación coloca a las víctimas en una posición social y académica difícil cuando se denuncia el incidente. El desafío se agrava aún más cuando las víctimas no reciben apoyo de sus familiares. Las respuestas recibidas se caracterizaron por la desconfianza y la ignorancia. La cultura del silencio, formada por el abuso de poder, la estigmatización y la desconfianza hacia la institución, convierte a los campus en lugares inseguros, especialmente para las mujeres y otros grupos vulnerables.

### Impactos Psicológicos en las Víctimas

La violencia sexual tiene consecuencias psicológicas graves y duraderas para las sobrevivientes. En el Campus A, una sobreviviente que sufrió abuso sexual por parte del “hermano mayor” sentía que “ya no era pura” y se preocupaba por el futuro. Lloraba y relató sentir que se habían violado su dignidad y su integridad moral. En el Campus A también se produjo un caso extremo de violencia sexual, en el que una estudiante sufrió abuso sexual repetido por parte de su supervisor. Debido a la presión psicológica sostenida, desarrolló una depresión grave, se autolesionó cortándose los brazos siguiendo un patrón específico y realizó varios intentos de suicidio. Su estado mental, gravemente desestabilizado, le impedía sentir dolor físico cuando se le trataban las heridas, lo que sugiere que el trauma había superado el umbral de la percepción física.

En el Campus B, la violencia sexual provocó importantes repercusiones psicológicas en las víctimas. Los funcionarios de la unidad de atención que trataron y ayudaron a las víctimas en su recuperación informaron que la mayoría de las personas cayeron en depresión y se suicidaron. Por lo tanto, se requiere apoyo psicológico intensivo para una recuperación efectiva. Los funcionarios de la unidad de atención a la violencia sexual también se enfocaron principalmente en abordar la recuperación psicológica profunda del trauma.

Otro impacto de la violencia sexual en las víctimas es la disminución del rendimiento académico, como lo experimentó una estudiante del Campus B. No tenía motivación para continuar con el proceso de supervisión de su tesis debido a su renuencia a reunirse con el supervisor, quien la había acosado sexualmente. Según la entrevista con el profesional de salud del campus, se informó que la estudiante padecía estrés agudo, náuseas persistentes y síntomas psicosomáticos. La intervención física y médica sería inadecuada, ya que la necesidad principal era la rehabilitación psicológica. La causa subyacente de su condición era el trauma psicológico debido al acoso sexual sufrido.

Las sobrevivientes de violencia sexual tienden a aislarse de la vida social. Estas personas suelen alejarse de las actividades académicas en el campus, evitan los espacios públicos y se sienten incómodas al interactuar con el sexo opuesto. En casos más leves, algunas sobrevivientes se sentían más cómodas cuando amigos cercanos las acompañaban en las actividades del campus. Esta experiencia traumática conduce a una sensación negativa de seguridad, tal como lo narró una informante:

*“A veces, la sobreviviente se muestra reacia a asistir a una reunión en el campus. Quedamos en encontrarnos en algún lugar fuera del campus, como una cafetería u otro sitio. Como tienen recuerdos traumáticos del campus, lo evitan”.* (AF)

Tras decidir denunciar los casos, las sobrevivientes de los dos campus experimentaron consecuencias psicológicas más complejas, que iban desde un trauma profundo hasta un sentimiento de impotencia debido a las respuestas inadecuadas. En el campus B, las sobrevivientes seguían sintiendo miedo porque los agresores permanecían en el campus mientras las denuncias no se habían resuelto. Incluso algunas sobrevivientes evitaron por completo ir al campus. En el campus A, algunas sobrevivientes sufrieron victimización secundaria, al tener que lidiar con la agresión inicial, el estigma social y la falta de apoyo sistémico. Algunas sobrevivientes se sentían “ya no puras” y persistentemente ansiosas por el futuro. Otras continuaron sufriendo acoso por parte de miembros del cuerpo docente a pesar de haber presentado denuncias formales.

Estos casos muestran un patrón constante de fracaso institucional en la prevención y el manejo de la violencia sexual en los campus. Las víctimas que están dispuestas a denunciar casos de violencia sexual en el campus a menudo experimentan impotencia y quedan atrapadas en un estado de miedo e incertidumbre.

## DISCUSIÓN

La violencia sexual en el campus tiene que ver con desequilibrios de poder y un apoyo institucional inadecuado para su prevención, como lo muestran una encuesta a 472 participantes y una entrevista en profundidad con 47 personas. La teoría de Foucault sobre las relaciones de poder explica cómo las estructuras jerárquicas —como la antigüedad, la autoridad religiosa y las relaciones dinámicas entre estudiantes y profesores— crean un espacio de explotación en el que los agresores utilizan posiciones dominantes para normalizar la violencia sexual. En este contexto, las relaciones de poder revelan una naturaleza relacional, dispersa y productiva^(21)^ que permite que la violencia sexual ocurra en el campus. La violencia se debe a la dominación individual, las redes de autoridad, las normas y la interacción social en el entorno académico. Foucault afirmó que el poder no solo era represivo, sino también productivo ^(22)^. En este marco, los campus producen un discurso que clasifica los comportamientos en “apropiados” e “inapropiados”. Esta categorización lleva a las personas a culpar a las sobrevivientes por la violación de las normas. Los estudios sobre la cultura de la vergüenza muestran que los casos de violencia sexual a menudo se silencian para proteger la reputación institucional, mientras que los agresores se salvan gracias a redes de poder^(23)^.

La alta vulnerabilidad ante la violencia sexual en los campus no puede separarse de las condiciones institucionales y de la implementación de políticas que aún son deficientes. Aunque algunos campus han establecido unidades de servicio integradas como parte de la prevención y la atención, la existencia de estas unidades en la práctica no ha contado con el pleno respaldo de las autoridades. En un caso, la unidad de servicio enfrentó limitaciones debido a la falta de apoyo financiero. Esto afectó el trabajo de atención, asistencia a las víctimas y educación de la comunidad académica. La situación sugiere que la existencia de políticas formales es insuficiente a menos que vaya acompañada de un compromiso institucional genuino. La percepción de algunos profesores de que la violencia sexual verbal es un hecho “normal” refleja la continua normalización de estos casos. Esta situación presenta desafíos adicionales para la unidad de servicios, ya que entre sus responsabilidades se incluyen la gestión de casos de violencia sexual y la lucha contra los conceptos erróneos dentro de la comunidad universitaria. Por lo tanto, se ve socavada la eficacia de las políticas universitarias, al tiempo que también disminuye la confianza de los estudiantes en el compromiso de la institución para abordar la violencia sexual.

Las respuestas lentas de las universidades ante los casos de violencia sexual aumentan el impacto en las sobrevivientes. Esta situación refleja lo que Holland y Cortina denominaron “traición institucional”, cuando una institución no cumple con su función de protectora^(24)^. Las universidades suelen priorizar su reputación al ocultar casos de violencia sexual o proteger a los agresores. Estas prácticas concuerdan con el concepto de “pacto patriarcal”^(25)^, en el que las mujeres se ven obligadas a negociar con un sistema que las pone en desventaja. En otro estudio^(26)^, la renuencia a presentar denuncias formales por temor a ser culpadas, ignoradas o acusadas de dañar la reputación de la institución constituye evidencia de la institucionalización de la cultura patriarcal en las políticas universitarias.

Este estudio demostró empíricamente el fracaso de los campus para generar confianza entre las sobrevivientes (Figura 2), ya que solo el 4% denunció experiencias de violencia sexual ante la unidad de atención. Mientras tanto, la divulgación informal se realizaba con frecuencia al hablar con los padres, amigos y la pareja. Este resultado concuerda con estudios previos, en los que las sobrevivientes tienden a preferir la divulgación informal en lugar de la denuncia formal ante la policía, las unidades de atención del campus u otras instituciones autorizadas que brindan servicios. La decisión puede verse influida por varios factores, entre ellos el temor a que se culpe a la víctima, la percepción de una reacción exagerada, el estigma social, las relaciones cercanas con los agresores y las experiencias negativas relatadas por otras sobrevivientes que previamente revelaron sus casos^(27)^. La revelación informal suele realizarse a través de conversaciones graduales y selectivas^(28)^. Este estudio también encontró que las diferentes formas de violencia sexual no se habían socializado lo suficiente entre la comunidad universitaria.

Las feministas amplían el análisis de Foucault al enfatizar que el control sobre los cuerpos femeninos es el núcleo del patriarcado^(29)^. En el contexto del entorno universitario, el control sobre el cuerpo femenino suele legitimarse mediante un discurso moral sesgado por el género. Este resultado quedó demostrado en el presente estudio, en el que varios factores percibidos como contribuyentes a la violencia sexual incluyeron la “ropa sexy”, la “vergüenza corporal” y las referencias a “mujeres coquetas”. Por el contrario, las acciones de los agresores se justificaban mediante referencias a las “citas”, la “promiscuidad”, la “fe débil”, la “lujuria” y el “estilo de vida”. Estos resultados coincidieron con otros estudios previos, en los que la violencia sexual a menudo se racionalizaba mediante la idea de que los hombres eran inherentemente dominantes, agresivos e incapaces de controlar sus deseos sexuales^(30)^.

Con base en la descripción anterior, este estudio destaca otro aspecto importante: la creciente disposición de las mujeres a dar a conocer sus experiencias de violencia sexual. Este valor no surgió de manera espontánea, sino que contó con el respaldo de redes de compañeras, organizaciones estudiantiles y unidades de atención a la violencia sexual. Este apoyo colectivo fortaleció su posición para enfrentar el trauma y les brindó un espacio seguro para denunciar los casos sin temor al estigma. Sin embargo, la falta de un apoyo institucional adecuado hizo que las voluntarias de las unidades de atención se sintieran aisladas en sus esfuerzos por abordar los casos de violencia sexual. En el Campus B, el proceso de redacción de los Procedimientos Operativos Estándar (POE) para la prevención tomó mucho tiempo debido a la falta de una comprensión adecuada de los indicadores en forma de abuso verbal. Otro estudio sistemático señala que los funcionarios o estructuras del campus que obstaculizan los programas de prevención constituyen una especie de traición institucional^(18)^.

## CONCLUSIÓN

En conclusión, la violencia sexual en los campus adoptó diversas formas, que van desde el abuso verbal, físico y digital hasta la coacción, con una alta prevalencia. El trauma genera graves consecuencias psicológicas para las sobrevivientes, específicamente para las mujeres que decidieron alzar la voz sobre sus experiencias. Algunas de las consecuencias psicológicas son la ansiedad prolongada, la pérdida de autoestima, la depresión grave y los intentos de suicidio. Se demostró que la asistencia psicosocial brindada por las unidades de servicio para la prevención de la violencia sexual en los campus A y B fue de vital importancia para la salud mental. Sin embargo, la asistencia religiosa y legal proporcionada por los campus fue insuficiente. Este estudio también confirmó que las dinámicas de poder, las respuestas lentas de las instituciones y el estigma social, en conjunto, generaron problemas psicológicos más complejos. Ha aumentado el número de mujeres que tienen el valor de hablar sobre sus experiencias de violencia sexual. Este valor cuenta con el apoyo de redes de pares, organizaciones estudiantiles y las unidades de servicio de los campus.

Las implicaciones indican que los campus deben fortalecer más a fondo el sistema de protección y manejo de la violencia sexual, centrándose en el mecanismo de denuncia y el apoyo psicosocial. Por lo tanto, las instituciones de educación superior deben ofrecer servicios más integrales, como asesoramiento, asistencia legal y apoyo espiritual inclusivo, lo que permita que el proceso de recuperación se desarrolle de manera más segura y sostenible. El fortalecimiento de la normativa interna y la aplicación de sanciones claras y firmes se convierten en medidas importantes para evitar la impunidad de los agresores. Los resultados confirman la necesidad de integrar la educación en materia de igualdad de género, la ética relacional y la capacitación en sensibilidad en el proceso de aprendizaje. Es importante considerar este tema dadas las fuertes perspectivas moralistas y la normalización de la violencia sexual en la percepción de la comunidad universitaria. Además, es necesario fortalecer la colaboración intersectorial entre los campus, las unidades de servicio, las agencias colaboradoras y las organizaciones de la sociedad civil para desarrollar un sistema integral de prevención y recuperación.

Este estudio se llevó a cabo únicamente en dos universidades estatales, lo que limita su generalización al contexto educativo nacional. El predominio del análisis descriptivo cuantitativo reduce la capacidad de realizar inferencias causales sobre la recuperación a largo plazo de las sobrevivientes. Otra limitación fue que las informantes fueron seleccionadas entre las personas que denunciaron los casos. Por lo tanto, este estudio no tomó en cuenta las perspectivas de las sobrevivientes que decidieron guardar silencio debido al estigma social, las barreras institucionales y otras razones.

Estudios futuros podrían ampliar el alcance institucional al incluir universidades privadas y aplicar análisis longitudinales para rastrear sistemáticamente la recuperación de los sobrevivientes. Los métodos utilizados deberían integrarse con un enfoque inferencial para obtener una comprensión profunda de las respuestas institucionales, las estrategias de afrontamiento de los sobrevivientes y la cultura académica.

## DISPONIBILIDAD DE DATOS

Los participantes no dieron su consentimiento por escrito para que los datos se compartieran públicamente. Por lo tanto, los datos de respaldo no están disponibles debido a la naturaleza sensible del estudio.
